# A Methodology for the Statistical Calibration of Complex Constitutive Material Models: Application to Temperature-Dependent Elasto-Visco-Plastic Materials

**DOI:** 10.3390/ma13194402

**Published:** 2020-10-02

**Authors:** Juan Luis de Pablos, Edoardo Menga, Ignacio Romero

**Affiliations:** 1 IMDEA Materials Institute, Eric Kandel, 2, 28906 Getafe, Spain; juanluis.pablos@imdea.org; 2 Mechanical Eng. Department, Universidad Politécnica de Madrid, José Gutiérrez Abascal, 2, 28006 Madrid, Spain; 3 AIRBUS Operations S.L., John Lennon S/N, 28906 Getafe, Spain; edoardo.menga@airbus.com

**Keywords:** model calibration, sensitivity analysis, elasto-visco-plasticity, Gaussian process

## Abstract

The calibration of any sophisticated model, and in particular a constitutive relation, is a complex problem that has a direct impact in the cost of generating experimental data and the accuracy of its prediction capacity. In this work, we address this common situation using a two-stage procedure. In order to evaluate the sensitivity of the model to its parameters, the first step in our approach consists of formulating a meta-model and employing it to identify the most relevant parameters. In the second step, a Bayesian calibration is performed on the most influential parameters of the model in order to obtain an optimal mean value and its associated uncertainty. We claim that this strategy is very efficient for a wide range of applications and can guide the design of experiments, thus reducing test campaigns and computational costs. Moreover, the use of Gaussian processes together with Bayesian calibration effectively combines the information coming from experiments and numerical simulations. The framework described is applied to the calibration of three widely employed material constitutive relations for metals under high strain rates and temperatures, namely, the Johnson–Cook, Zerilli–Armstrong, and Arrhenius models.

## 1. Introduction

Modeling has become a very effective way to analyze, in a first instance, complex engineering problems. Almost the totality of engineers, either in academia or in the industry, claim to take benefit from these techniques, considering them to be irreplaceable for their work.

Though the reliability of models keeps constantly increasing, and therefore the trust placed on their predictions, there is still need for understanding the intrinsic uncertainties that affect simulation, for estimating their effect on predictions, and for developing efficient methodologies to reduce them in a cost-effective manner. In this respect, an interesting and promising approach has emerged in recent years. It consists of employing advanced statistical methods not only to assess the uncertainty in a model but also to guide the experimental campaign that needs to be carried out to feed the parameter calibration. One of these tools is Global Sensitivity Analysis (GSA), a very useful strategy when it comes to analyzing the influence of all the parameters participating in a model. Improving local techniques introduced in the 1980s [1], GSA methods were proposed much later to account for the influence of parameters in an overall and rigorous fashion [2].

**Figure 1:**
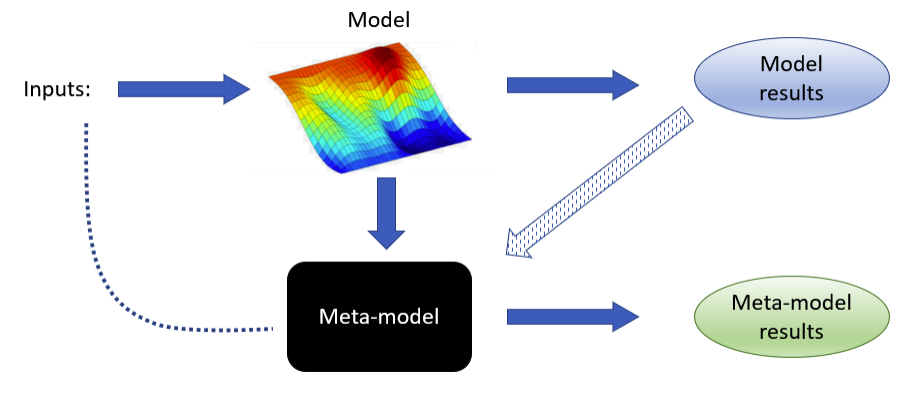
Meta-modeling construction process.

One important limitation of GSA techniques is that they require large amounts of simulated data as input. Numerical experiments obtained, for instance, through finite element (FE) simulations, demand a huge amount of computational resources that are often unavailable. A convenient remedy to this problem is to employ meta-models that provide reasonable approximations to the models’ response, but at a fraction of their computational cost. These types of models are built by sampling the original ones, as illustrated in Figure 1, and were originally proposed to optimize processes [3]. Initially known as Response Surface Models (RSM), they rapidly evolved and became emulators of computational codes at a very reduced cost. As a result, they have been utilized in a wide range of sensitivity analyses and applications [4,5,6].

There exist several families of meta-models. Some of the most commonly employed are the ones based on Kriging [7] and Radial Basis Functions (RBF) [8]. Both of them are generally accepted as good methods to efficiently capture trends associated with small data sets. Since they accurately adapt to available information, they must be re-calibrated when new inputs are provided [9]. The Bayesian approach, on the other hand, is a well-known technique that has been successfully employed in several scientific disciplines for parameter selection. See, for example, one of the very first generic applications, developed by Guttman [10], in which this inference procedure is already used to choose the best manufacturing parameters to make the widest possible population of fabricated items lie within the specified tolerance limits. More recent works have improved the Bayesian inference methodology (see, e.g., [11]).

Bayesian inference can be used systematically for the calibration of model parameters, taking into consideration the uncertainties due to the model itself, the experimental measurements, noise, etc. [12,13,14]. This approach has become relatively standard, not only providing optimized parameter values but a complete Gaussian distribution for them.

In this work, we will combine GSA with Bayesian calibration because we believe that this combination is extremely powerful for understanding computer models, and drawing as much information as possible from experiments, be them numerical or physical. This mix of techniques is not new, and similar ones have been considered in the past. For example, sensitivity analysis and Bayesian calibration were employed together in [15], trying to assess multiple sources of uncertainty in waste disposal models by considering independent and composite scenarios, obtaining predictive output distributions using a Bayesian approach, and later performing a variance-based sensitivity analysis. In addition, the work by [16] proposes a procedure to evaluate the sensitivity of the parameters and a posterior calibration of the most important ones applied to a model describing the chemical composition of mass of waters.

In the current article, we explore the use of GSA and Bayesian calibration for complex constitutive models, an application that has not been previously considered for this type of analysis and that can greatly benefit from it. More precisely, we study three fairly known constitutive material models suitable for metals subjected to extreme conditions, namely, Johnson–Cook [17], Zerilli–Armstrong [18], and Arrhenius-type [19] models. These are fairly complex constitutive relations that depend on a relatively large number of material parameters that need to be adjusted for each specific material and test range. The actual implementations of the three can be found in the publicly available material library MUESLI [20], and we have used them together with standard explicit finite element calculations.

The remainder of the article is structured as follows. In Section 2, we will outline the theoretical principles in which the statistical theory employed is based on, as well as the three constitutive material models used in the study. In Section 3, we describe the application of the presented framework to the analysis of Taylor’s impact test [21,22], an experiment often used to characterize the elastoplastic behavior of metals under high strain rates. The results of our investigation are reported in Section 4, providing insights for the three constitutive models. Finally, Section 5 collects the main findings and conclusions of the study.

## 2. Fundamentals

### 2.1. Global Sensitivity Analysis

Global Sensitivity Analysis (GSA) refers to a collection of techniques that allow identifying the most relevant variables in a model with respect to given Quantities of Interest (QoIs). They focus on apportioning the output’s uncertainty to the different sources of uncertain parameters [2] and define qualitative and quantitative mappings between the multi-dimensional space of stochastic variables in the input and the output. The most popular GSA techniques are based on the decomposition of the variance’s output probability distribution and allow the calculation of Sobol’s sensitivity indices.

According to Sobol’s decomposition theory, a function can be approximated as the sum of functions of increasing dimensionality and orthogonal with respect to the standard $L_{2}$ inner product. Hence, given a mathematical model y=f(x) with *n* parameters arranged in the input vector *x*, the decomposition can be expressed as:

(1) $$f\left(x_{1},\ldots ,x_{n}\right)=f_{0}+\sum_{i=1}^{n}f_{i}\left(x_{i}\right)+\sum_{1\leq i<j\leq n}f_{ij}\left(x_{i},x_{j}\right)+\cdots +f_{1,2,\ldots ,n}\left(x_{1},\ldots ,x_{n}\right),$$

where $f_{0}$ is a constant and $f_{\ldots }$ are functions with domains of increasing dimensionality. If we consider that *f* is defined on random variables $X_{i}∼U\left(0,1\right),i=1,\ldots ,n$, then the model output is itself a random variable with variance

(2) $$D=\mathrm{Var}\left[f\right]=\int_{R^{n}}f^{2}\left(x\right)\;dx-f_{o}^{2}.$$

Integrating Equation (1) and using the orthogonality property of the functions $f_{\ldots }$, we note that the variance itself can be decomposed in the sum

(3) $$D=\sum_{i=1}^{n}D_{i}+\sum_{1\leq i<j\leq n}D_{ij}+\cdots +D_{1,2,\ldots ,n}.$$

This expression motivates the definition of the Sobol indices

(4) $$S_{i_{1},\ldots ,i_{s}}=D_{i_{1},\ldots ,i_{s}}/D,$$

that trivially satisfy

(5) $$\sum_{i=1}^{n}S_{i}+\sum_{1\leq i<j\leq n}S_{ij}+\cdots +S_{1,2,\ldots ,n}=1.$$

This decomposition of the total variance *D* reveals the main metrics employed to assess the relevance of each parameter in the scatter of the quantity of interest *f*. The relative variances $S_{i}$ are referred to as the first order indices or main effects and gauge the influence of the *i*-th parameter on the model’s output. The total effect or total order sensitivities associated with the i—parameter, including its influence when combined with other parameters, is calculated as

(6) $$S_{T_{i}}=\sum_{I_{i}}D_{i_{1},\ldots ,i_{s}},\;I_{i}=\left\{\left(i_{1},\ldots ,i_{s}\right):\exists k,1\leq k\leq s,i_{k}=i\right\}.$$

The widespread use of the main and total effects as sensitivity measures is due to the relative simplicity of the formulas and algorithms that can be employed to calculate or approximate them ([2], Chapter 4). Specifically, the number of simulations required to evaluate these measures is $N_{s}\left(n+2\right)$, where $N_{s}$ is the so-called base sample, a number that depends on the model complexity varying from a few hundreds to several thousands, and *n* is, as before, the number of parameters of the model (we refer to ([2], Chapter 4) for details on these figures). To calculate sensitivity indices, it proves essential to first create a simple meta-model that approximates the true model, because a limited set of runs is supposed to suffice for building a surrogate that, demanding far fewer computational resources, can then be run a large number of times to complete the GSA.

### 2.2. Meta-Models

A meta-model is a model for another model. That is, a much-simplified version of a given model that can provide, however, similar predictions as the original one for the same values of the parameters. In this work, we restrict our study to linear meta-models. To describe them, let us assume that the model we are trying to simplify depends on $N_{p}$ parameters and we denote the quantity of interest, assumed for simplicity to be a scalar, as *y*. For any collection of parameters $x\in R^{N_{p}}$, we define $\hat{y}\left(x\right)$ to be the corresponding value of the quantity of interest and slightly abusing the notation we write

(7) $$\hat{y}\left(x\right)=\left[\hat{y}\left(x_{1}\right),\hat{y}\left(x_{2}\right),\ldots ,\hat{y}\left(x_{N}\right)\right]\;,$$

where *N* is the number of samples and $x=[x_{1},x_{2},\ldots ,x_{N}]$ is an array of *N* samples of the parameters. Then, a meta-model is a function $\hat{Y}:R^{N_{p}}\to R$ that approximates $\hat{y}$ and is of the form

(8) $$\hat{Y}\left(x\right):=\sum_{k=1}^{N_{k}}\eta_{k}h_{k}\left(x\right)\;.$$

In this equation, and later, $h_{k}$ are the kernels of the approximation and $\eta_{k}$ refer to the weights. Abusing slightly the notation again, we express the relation between the model and its meta-model as

(9) $$\hat{y}\left(x\right)\approx \hat{Y}\left(x\right)=\sum_{k=1}^{N_{k}}\eta_{k}h_{k}\left(x\right),$$

or in compact form,

(10) $$\hat{y}\left(x\right)\approx \hat{Y}\left(x\right):=H\left(x\right)\eta ,$$

where *H* is the so-called kernel matrix that collects all the kernel functions.

The precise definition of a meta-model depends, hence, on the number and type of kernel functions $h_{k}$ and the value of the regression coefficients $\eta_{k}$. Given an a priori choice for the kernels, the weights can be obtained from a set of model evaluations $\hat{y}\left(x\right)$ employing a least-squares minimization. Given, as before, an array of sample parameters x and their model evaluation $\hat{y}\left(x\right)$, the vector η can be calculated in closed form with the solution of the normal equations to the approximation. That is,

(11) $$\eta ={\left(H{\left(x\right)}^{T}H\left(x\right)\right)}^{-1}H{\left(x\right)}^{T}\hat{y}\left(x\right).$$

As previously indicated, the kernel functions belong to a set that must be selected a priori. In the literature, several classes of kernel have been proposed for approximating purposes and in this work we select anisotropic Radial Basis Functions (RBF). This type of kernel has shown improved accuracy as compared with standard RBF, particularly when only a limited set of model evaluations is available [23].

A standard RBF is a map $K:R^{N_{p}}\times R^{N_{p}}\to R$ of the form

(12) K(x,z)=k(r(x,z)),

where k:R→R is a monotonically decreasing function and r(x,z):=|x−z| is the Euclidean distance. The anisotropic radial kernels redefine the function *K* to be of the form

(13) $$K\left(x,z\right)=\exp\left[-\epsilon \sum_{i=1}^{N_{d}}\gamma_{i}^{2}{\left(x_{i}-z_{i}\right)}^{2}\right]=\exp\left[-\epsilon {\left(x-z\right)}^{T}\Gamma \left(x-z\right)\right],$$

where $\Gamma =\mathrm{diag}(\gamma_{1}^{2},\ldots ,\gamma_{N_{d}}^{2})$ is a diagonal, positive definite matrix that scales anisotropically the contribution of each direction in the difference x−z, and ϵ>0 is the shape parameter of the kernel.

### 2.3. Bayesian Inference and Gaussian Processes

Bayesian inference is a mathematical technique used to improve the knowledge of probability distributions extracting information from sampled data [24]. It is successfully employed for a wide variety of applications in data science and applied sciences. For instance, it has been used in conjunction with techniques such as machine learning and deep learning in fields like medicine [25], robotics [26], earth sciences [27], and more. In this article, we employ Bayesian methods to find the optimal value of model parameters as a function of prior information and observed data. More specifically, we are concerned with model calibration and using it in combination with meta-models to obtain realistic parameter values for complex constitutive relations and their uncertainty quantification, with an affordable computational cost.

Some of the most robust techniques for calibration are based on non-parametric models for nonlinear regression [28]. Here, we will employ Gaussian processes to represent in an abstract fashion the response of a simulation code to a complex mechanical problem employing the material models that we are set to study. We summarize next the main concepts behind these processes.

A Gaussian process is a set of random variables such that any finite subset of them has a Gaussian multivariate distribution [28]. Such a process is completely defined by its mean and covariance, which are functions. If the set of random variables is indexed by points $x\in X\subset R^{d}$, then when the random variables have a scalar value f(x), the standard notation employed is

(14) $$f\left(x\right)∼\mathrm{GP}\left(m\left(x\right),k\left(x,x^{\prime }\right)\right),$$

where m:X→R and k:X×X→R are, respectively, the mean and covariance. The mean function can be arbitrary, but the covariance must be a positive function. Often these two functions are given explicit expressions depending on hyperparameters. In simple cases, the average is assumed to be zero, but often it is assumed to be of the form

(15) $$m\left(x\right)=g{\left(x\right)}^{T}\beta \;,$$

where $g:R^{d}\to R^{g}$ is a vector of known basis functions and $\beta \in R^{g}$ is a vector of basis coefficients. The choice of the covariance function is the key aspect that determines the properties of the Gaussian process. It is often selected to be stationary, that is, depending only on a distance $d=\hat{d}\left(x,x^{\prime }\right)$. In particular, we will employ a covariance function such as

(16) $$c\left(x,x^{\prime }\right)=\sigma^{2}r\left(x,x^{\prime }\right),$$

where $\sigma^{2}$ is a variance hyperparameter and $r:R^{d}\times R^{d}\to R$ has been chosen as the Màtern $C_{5/2}$ function, an isotropic, differentiable, stationary kernel commonly used in statistical fitting that is of the form

(17) $$r\left(x,x^{\prime }\right)=\left(1+\sqrt{5}\frac{{\hat{d}}^{2}\left(x,x^{\prime }\right)}{\psi }+5\frac{{\hat{d}}^{2}\left(x,x^{\prime }\right)}{3\psi^{2}}\right)\exp\left[-\sqrt{3}\frac{{\hat{d}}^{2}\left(x,x^{\prime }\right)}{\psi }\right],$$

that uses a length-scale hyperparameter ψ. For the Gaussian process described, the collection of hyperparameters can be collected as $\chi =(\beta ,\sigma^{2},\psi )$.

Let us now describe in which sense Bayesian analysis can be used for model calibration. The value of a computer model depends on the value of some input variables x that are measurable, and some parameters t that are difficult to determine because they are not directly measurable. Let us assume that t=θ is the true value of the parameters, which is unknown and we would like to determine based on available data. Given some input variable x, a physical experiment of the problem we want to model will produce a scalar output *z* and it will verify

(18) z=η(x,θ)+δ(x)+ε(x).

In this equation, η(x,θ) is the value of the computer model evaluated at the input variable x and the true parameter θ, δ(x) is the so-called model inadequacy and ε(x) is the observation error. This last term can be taken to be a random variable with a Gaussian probability distribution $N(0,\lambda^{2})$. The functions η and δ are completely unknown so we can assume them to be Gaussian processes with hyperparameters $\chi_{\eta }$ and $\chi_{\delta }$, respectively.

If $\theta ,\chi_{\eta },\chi_{\delta },\lambda^{2}$ were known, we could study the multivariate probability distribution of the output *z* using Equation (18) for any set of inputs $\left(x_{1},x_{2},\ldots ,x_{s}\right)$. However, we are interested in solving the inverse problem: we have a set of experimental and computational data and we would like to determine the most likely probability distribution for θ and the hyperparameters, a problem that can be effectively addressed using Bayes’ theorem.

Bayes’ theorem states that, given a prior probability for the parameters $\left(\theta ,\chi_{\eta },\chi_{\delta },\lambda^{2}\right)$ indicated as $p(\theta ,\chi_{\eta },\chi_{\delta },\lambda^{2})$, the posterior probability density function for these parameters after obtaining the data Δ is

(19) $$p\left(\theta ,\chi_{\eta },\chi_{\delta },\lambda^{2}|\Delta \right)\propto p\left(\theta ,\chi_{\eta },\chi_{\delta },\lambda^{2}\right)\;p\left(\Delta |\theta ,\chi_{\eta },\chi_{\delta },\lambda^{2}\right)\;.$$

The prior for the parameters and hyperparameters can be taken as Gaussian, or any other probability distribution that fits our initial knowledge. Assuming that the parameters and hyperparameters are independent, we have in any case that

(20) $$p\left(\theta ,\chi_{\eta },\chi_{\delta },\lambda^{2}\right)=p\left(\theta \right)\;p\left(\chi_{\eta },\chi_{\delta },\lambda^{2}\right)\;.$$

In addition, since Equation (18) indicates that the output is the sum of three random variables with Gaussian distributions, *z* itself is a Gaussian.

To apply Bayes’ theorem, it remains to calculate the likelihood $p(\Delta |\theta ,\chi_{\eta },\chi_{\delta },\lambda^{2})$. To this end, the hyperparameters of η and δ are collected together with the observation error ε and we assume that the conditioned random variable $\Delta |\theta ,\chi_{\eta },\chi_{\delta },\lambda^{2}$ has a normal probability distribution of the form

(21) $$N\{E\left[\Delta |\theta ,\chi_{\eta },\chi_{\delta },\lambda^{2}\right],\mathrm{Var}\left[\Delta |\theta ,\chi_{\eta },\chi_{\delta },\lambda^{2}\right]\}.$$

Here, E[·] and Var[·] refer to the expectation and variance, respectively. Finally, in order to obtain the segregated posterior probability density of the parameters p(θ|Δ), we should integrate out $\chi_{\eta },\chi_{\delta }$ and $\lambda^{2}$; but, due to the high computational cost involved, this is typically done using Monte Carlo methods [29]. Details of this process fall outside the scope of the present work and can be found in standard references [12].

### 2.4. Material Models

In Section 4, a sensitivity and calibration procedure is applied to three relatively complex material models employed in advanced industrial applications. Here, we summarize them, listing all the parameters involved in their description. The remainder of the article will focus on ranking the significance of these parameters, their influence in the predictions, and the determination of their probability distributions.

#### 2.4.1. Johnson–Cook Constitutive Relations

The Johnson–Cook (JC) constitutive model [17] is commonly used to reproduce the behavior of metals subjected to large strains, high strain rates, and high temperatures. It is not extremely accurate in all ranges of strain rates and temperatures, but it is simple to implement, robust, and, as a result, has been employed over the years in a large number of applications [30,31,32].

Johnson–Cook’s model is a $J_{2}$ classical plasticity constitutive law in which the yield stress $\sigma_{y}$ is assumed to be of the form:

(22) $$\sigma_{y}=\left(A+B\varepsilon_{p}^{n}\right)\left(1+C\log{\dot{\varepsilon }}_{p}^{*}\right)\left(1-T^{*m}\right),$$

where $\varepsilon_{p}$ refers to the equivalent plastic strain. The first term in expression (22) accounts for the quasistatic hardening, including the initial yield stress, *A*, and the constants representing the strain hardening, *B*, and the exponent *n*. The second term in (22) is related to the hardening due to strain rate effects, containing the strain rate constant, *C* and also the dimensionless plastic strain rate, ${\dot{\varepsilon }}^{*}=\frac{{\dot{\varepsilon }}_{p}}{{\dot{\varepsilon }}_{p0}}$, where ${\dot{\varepsilon }}_{p0}$ is a reference plastic strain rate, often taken to be equal to 1. Finally, the third term accounts for the effects of temperature, including the thermal softening exponent, *m*, and also the so-called “homologous temperature”

(23) $$T^{*}=\frac{T_{exp}-T_{room}}{T_{melt}-T_{room}}.$$

Here, $T_{exp}$ is the experimental temperature at which the material is being modeled, $T_{melt}$ is the melting temperature of the material and $T_{room}$ is the ambient temperature.

#### 2.4.2. Zerilli–Armstrong Constitutive Relations

The Zerilli–Armstrong (ZA) model [18] was conceived as a new approach to the metal plasticity modeling, using a finite deformation formulation based on constitutive relations related to the physical phenomenon of dislocation mechanics, in contrast to other purely phenomenological constitutive relations, such as the previously described Johnson–Cook model. These relations have been proved to be well suited for the modeling of the response of metals to high strains, strain rates, and temperatures. Its numerical implementation, although more complicated than the JC model, is still relatively simple, justifying its popularity.

The ZA relations were developed in order to respond to the need of a physical-based model that could include the high dependence of the flow stress of metals and alloys on dislocation mechanics. For instance, aspects like the grain size, thermal activation energy, or the characteristic crystal unit cell structure have a dramatic effect in the plastic response of these materials, according to experimental data. Hence, the ZA model is still a $J_{2}$ plasticity model in which the yield stress becomes a function of strain, strain rate, and temperature, but with their relative contributions weighted by constants that have physical meaning. The yield stress is assumed to be

(24) $$\sigma_{y}=\left(C_{1}+C_{2}\varepsilon_{p}^{\frac{1}{2}}\right)\exp\left(-C_{3}T+C_{4}T\log\dot{\varepsilon_{p}}\right)+C_{5}\varepsilon_{p}^{n}+kl^{-\frac{1}{2}}+\sigma_{G}.$$

In this relation, $C_{1},C_{2},C_{3},C_{4},C_{5},k,\sigma_{G},l$ are constants. The constants $\sigma_{G},k,l$ represent, respectively, the contributions to the yield stress due to solutes, the initial dislocation density, and the average grain diameter. The remaining constants are selected to distribute the contribution to the hardening of the plastic strain, its rate, and the temperature. Based on the crystallographic structure of the metal under study, some of the constants $C_{i}$ will be set to zero. For example, fcc metals such as copper will have $C_{1}=C_{5}=0$. Iron and other bcc metals will be represented with equations that have $C_{2}=0$. These differences are mainly based on the physical influence of the effects of strain on each type of structure, which is especially dominant when it comes to modeling fcc metals, whereas the strain-rate hardening, thermal softening, and grain size have a greater effect on bcc metals.

#### 2.4.3. Arrhenius-Type Model Constitutive Relations

Last, we consider an Arrhenius-type (AR) constitutive model [19], a strain-compensated equation aiming to reproduce the behavior of metals at high temperature. As in the previous constitutive laws, the AR model is a classical $J_{2}$ plasticity model with an elaborated expression for the yield stress $\sigma_{y}$. In this case, it is defined as

(25) $$\sigma_{y}=\frac{1}{\alpha (\varepsilon_{p})}\sinh^{-1}{\left(\frac{Z(\varepsilon_{p},\dot{\varepsilon_{p}},T)}{A(\varepsilon_{p})}\right)}^{1/n},$$

where α:R→R and A:R→R are two functions employed to represent the influence of the plastic strain on the response and *n* is a material exponent. On the other hand, $Z:R\times R\times R^{+}\to R$ is the so-called Zener–Holloman function, accounts for the effects of strain rate $\dot{\varepsilon_{p}}$ and temperature *T*, and is defined as

(26) $$Z\left(\varepsilon_{p},\dot{\varepsilon_{p}},T\right):=\dot{\varepsilon_{p}}\exp\left(\frac{Q(\varepsilon_{p})}{RT}\right),$$

where *R* is the universal gas constant and Q:R→R is the activation energy, assumed to be a third-order polynomial.

The scalar functions that enter the definition of the yield function are thus α,A and *Q*. The three are defined parametrically as

(27) $$\begin{array}{cc} \alpha (\varepsilon_{p})\; & =\alpha_{0}+\alpha_{1}\varepsilon_{p}+\alpha_{2}\varepsilon_{p}^{2}+\alpha_{3}\varepsilon_{p}^{3},\\ Q(\varepsilon_{p})\; & =Q_{0}+Q_{1}\varepsilon_{p}+Q_{2}\varepsilon_{p}^{2}+Q_{3}\varepsilon_{p}^{3},\\ A(\varepsilon_{p})\; & =\exp\left[A_{0}+A_{1}\varepsilon_{p}+A_{2}\varepsilon_{p}^{2}+A_{3}\varepsilon_{p}^{3}\right], \end{array}$$

where $\alpha_{0},\ldots ,\alpha_{3}$, $Q_{0},\ldots ,Q_{3}$, and $A_{0},\ldots ,A_{3}$ are material constants determined experimentally. Depending on the author, these three functions might adopt slightly different forms leading to potentially higher accuracy at the expense of more difficulties for their calibration.

## 3. Application

**Figure 2:**
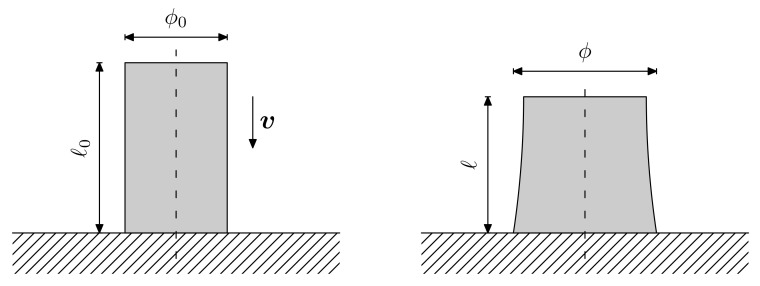
Schematic of Taylor’s impact test.

The methodology presented in Section 2 is applied now to a relevant example in mechanics of deformable solids, namely, Taylor’s impact test [21,22]. In what follows, we will study the calibration of the three material models of Section 2.4 based on the outputs obtained from this well-known test that consists of a high-velocity impact of a metallic anvil onto a rigid wall. As illustrated in Figure 2, the impact creates irrecoverable deformations in the anvil that, due to the symmetry of the problem, can be macroscopically quantified by measuring the changes in the diameter and length of the impactor.

**Figure 3:**
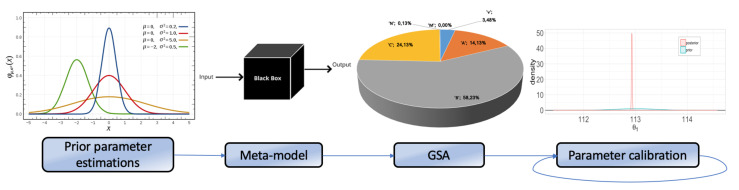
Iterative process for a two-stage approach of screening and calibration of model parameters.

Figure 3 illustrates the procedure advocated for our numerical analysis: starting from a prior distribution for the material parameters, a meta-model of Taylor’s impact test is constructed based on anisotropic RBF. The meta-model, once completed, is cheap to run and can be used to perform sensitivity analyses and to update, via Bayesian calibration, the probability distribution of the original parameters. If deemed necessary, the latter probability distribution can be reintroduced in the Bayesian calibration, this time as prior, as illustrated in Figure 3, until the parameter distribution converges to an (almost) stationary function. In theory, one could use the posterior probabilities to start the whole process, helping to build a better meta-model that will be later employed in the GSA and calibration. This route, however, might be too expensive in real life applications.

To build the meta-model, five impact velocities are selected over a typical range of Taylor’s bar experiments: namely, 200, 230, 260, 290, and 320 m/s. Then, the different tests for each impact velocity are simulated considering a Cr-Mo steel as the anvil’s material. Each impact velocity point consists of 612 simulations for the Johnson–Cook and Zerilli–Armstrong models, and 1800 for the Arrherius-type, since the latter involves a larger number of material parameters and requires more data in order to get reliable levels of accuracy when constructing the meta-models. The parameters fed to the simulations have been sampled from uniform distributions centered at nominal values taken from the literature [19,33] with ±10% ranges, varying them according to a Low Discrepancy Design method (LDD), or Sobol sequence [34]. The latter is obtained with a deterministic algorithm that subdivides each dimension of the sample space into $2^{N}$ points, while ensuring good uniformity properties.

The QoIs selected for the meta-model are ΔR and ΔL; that is, the changes in radius and length of the anvil after impact. Using the methods described in Section 2.2, an RBF-based meta-model is obtained for each material and impact velocity. The meta-models now serve as the basis for the Global Sensitivity Analysis that will identify the most significant parameters in each model, ruling out from the Bayesian calibration those whose influence on the QoIs is relatively small. Finally, for each of the material models, a full Bayesian analysis will be done based on the concepts of Section 2.3, providing a fitted Gaussian process per model and QoI. This last step demands standard but cumbersome operations and has been performed using a freely available R package [35].

**Figure 4:**
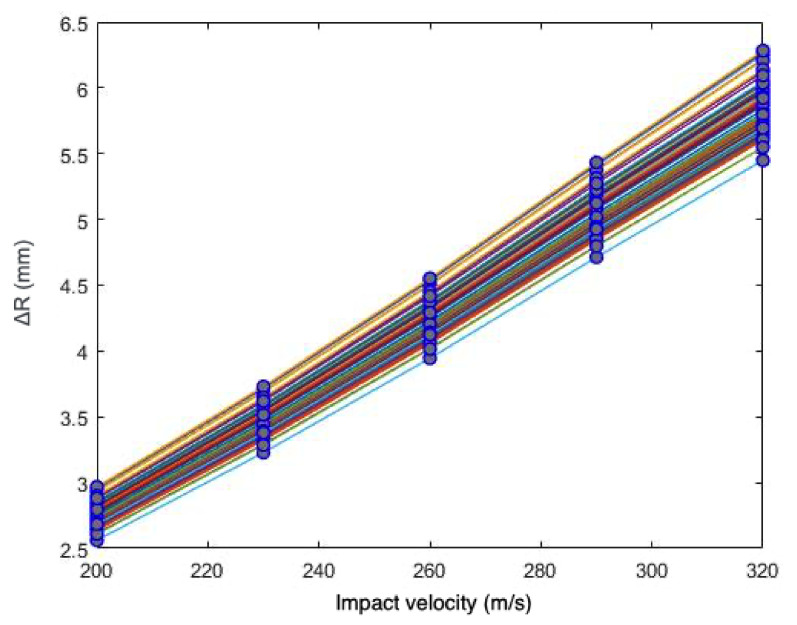
Linear interpolation of meta-model predictions of ΔR for the Johnson–Cook constitutive relation. Each piecewise linear interpolation connects predictions with the same model parameters.

**Figure 5:**
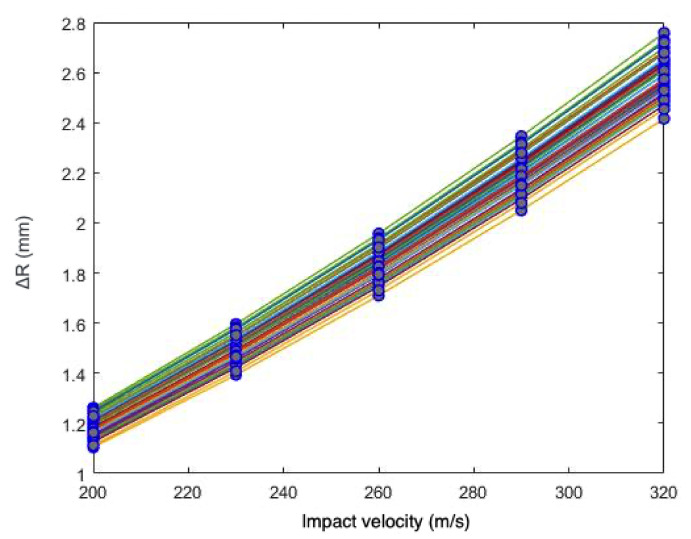
Linear interpolation of meta-model predictions of ΔR for the Zerilli–Armstrong constitutive relation.

**Figure 6:**
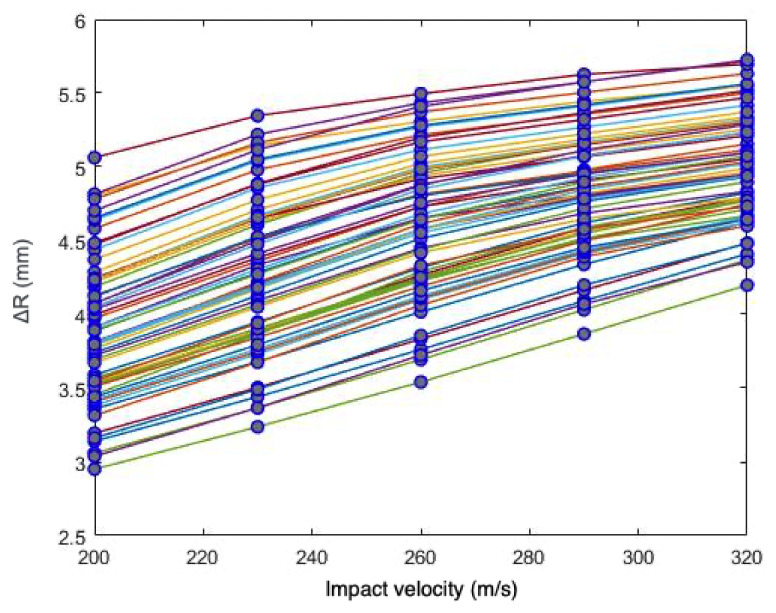
Linear interpolation of meta-model predictions of ΔR for the Arrhenius-type constitutive relation.

To complete the Bayesian calibration, we need meta-model predictions for arbitrary velocities of the impactor. Since the available meta-models are only defined for five selected velocities, we will interpolate linearly their predictions for the QoI at any intermediate velocity (see Figure 4, Figure 5 and Figure 6). This strategy will speed-up the generation of data for the Bayesian analysis. To validate it, we will first confirm that the error made by this interpolation is negligible. For that, we will compare solutions obtained with FE simulations at arbitrary velocities of the anvil against interpolated meta-model predictions.

Once accepted, this strategy for combining meta-models will result in an extremely cheap source of simulated data that will be used to study the material models. For each of the latter, the fitting data will consist of $n_{1}=20$ sets of observed points $\Delta_{1}=\left\{x_{1},\ldots ,x_{n_{1}}\right\}$, plus $n_{2}=500$ sets of computational outputs, derived from the meta-models interpolation, $\Delta_{2}=\left(x_{1}^{\prime },t_{1}\right),\ldots ,\left(x_{n_{2}}^{\prime },t_{n_{2}}\right)$, where $x_{i}$ and $x_{i}^{\prime }$ are the experimental impact velocities and interpolated impact velocities acting as the variable input, respectively, while $t_{i}$ are the parameter inputs to the meta-model. To assess the results of the meta-models interpolation, the FE cases against which are to be compared will be generated employing the same parameter inputs $t_{i}$ and impact velocities $x_{i}^{\prime }$.

In this work, we have chosen to calibrate CrMo steel because the parameters for the JC, ZA, and ARR models could be found in the literature for this material. However, no experimental measurements are available for Taylor tests with anvils of this material. Hence, we follow an alternative avenue to obtain data, one that is often employed in statistical analyses [36,37]. The idea is to generate data from finite element simulations (20 in our procedure) using a fixed material model with nominal parameters, exploring all impact velocities and adding white Gaussian noise to all the measured QoIs, consistent with Equation (18).

To complete the problem definition, it remains to choose prior probability distributions for the complete set of material parameters θ, the variance of the global observation error $\lambda^{2}$, and the hyperparameters $\chi_{\delta }$ of the discrepancy function. Table 1 and Table 2 describe the probability distributions chosen for each parameter in the three models and the references employed for their choice.

## 4. Results

We now present the results of the GSA analyses, the meta-models interpolation and calibration procedure for the three material models described in Section 2.4 based on the results obtained from the experiments of Taylor’s anvil impact. These are obtained from the RBF meta-model whose construction is detailed in Section 2.2 and Section 3.

### 4.1. Sensitivity Analysis

**Figure 7:**
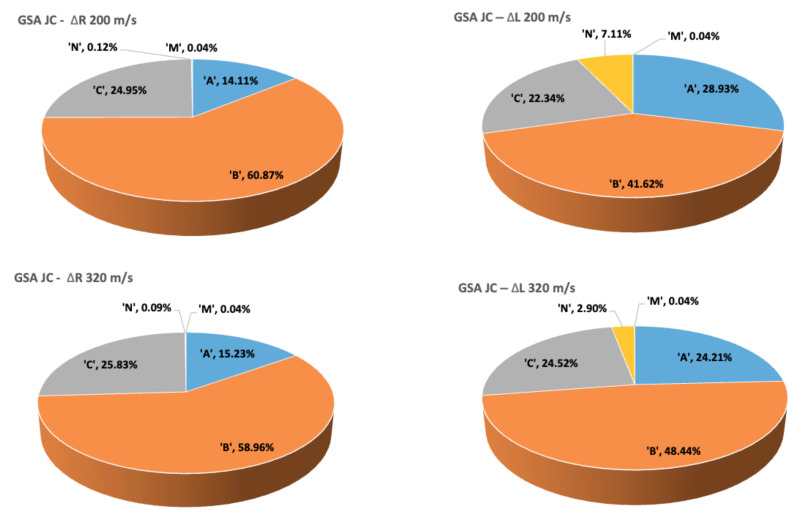
Global Sensitivity Analysis (GSA) results for the Johnson–Cook (JC) model considering ΔR and ΔL at 200 and 320m/s.

**Figure 8:**
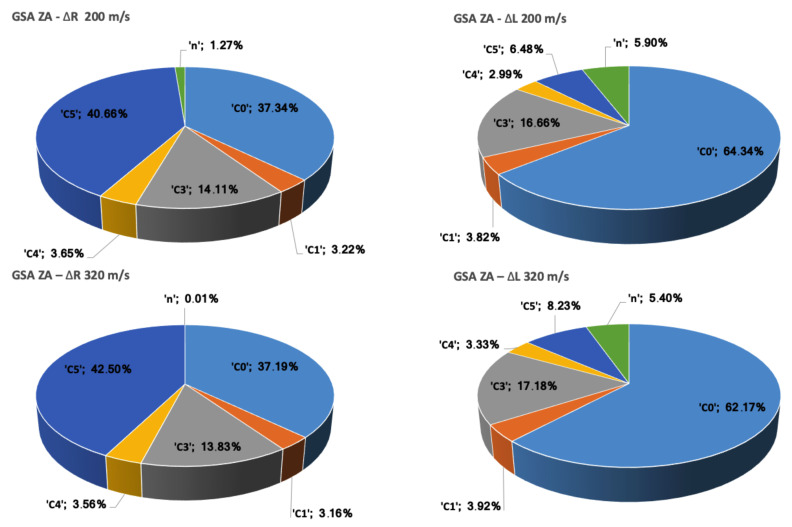
GSA results for the Zerilli–Armstrong (ZA) model considering ΔR and ΔL at 200 and 320m/s.

**Figure 9:**
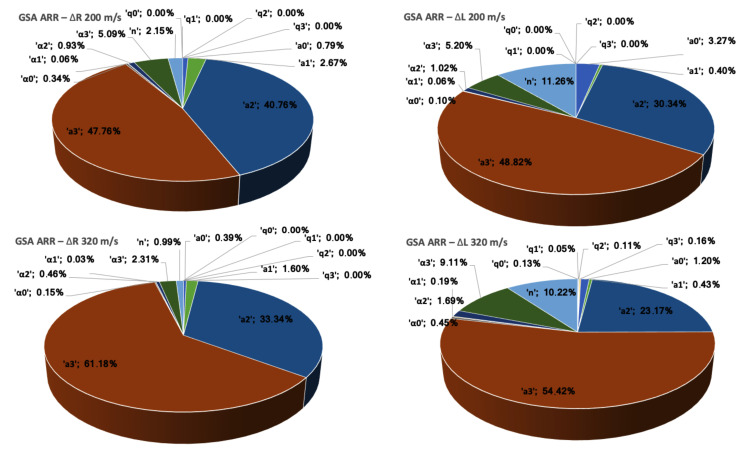
GSA results for the Arrhenius-type model considering ΔR and ΔL at 200 and 320m/s.

First, we present the results of the sensitivity analyses, as summarized in the pie charts of Figure 7, Figure 8 and Figure 9. For each of the three material models, these figures depict the contributions, at two impact velocities, of the parameters to global variance, considering independently the two QoIs: namely, the increments in anvil’s radius and length.

In all the three models, the pie charts expressing the parameters’ influence are slightly different, as expected from a complex experiment. However, the most significant result of the analysis performed is that the most influential parameters of each material model coincide in the four sensitivity figures.

To proceed, we identify for each material model the smallest set of parameters whose combined influence accounts for at least 90% of the total QoI variance in all the tests performed and we summarize these findings in Table 3. These results are useful in two ways. First, they simplify the ensuing Bayesian calibration, limiting the number of hyperparameters for the Gaussian processes and the computations involved in the likelihood calculations. Second, from a quantitative point of view, it can be employed by users of material models in numerical simulations to reveal the most influential parameters in the three laws considered, where most of the calibration efforts should be placed, irrespective of the methodology followed to this end.

### 4.2. Linear Interpolation of Meta-Models

**Figure 10:**
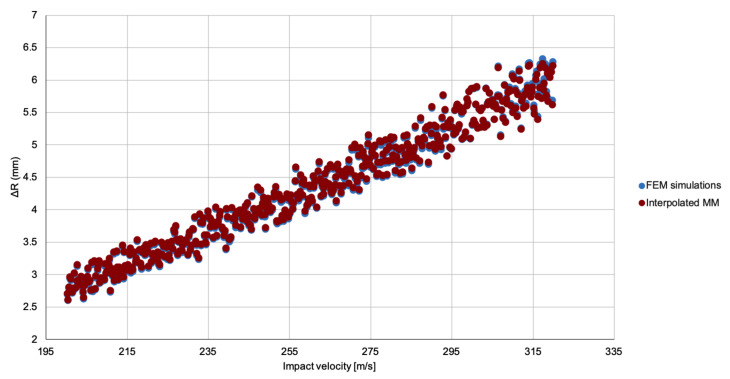
Comparison of meta-models interpolated results and FEM simulations, considering ΔR for the Johnson–Cook model.

**Figure 11:**
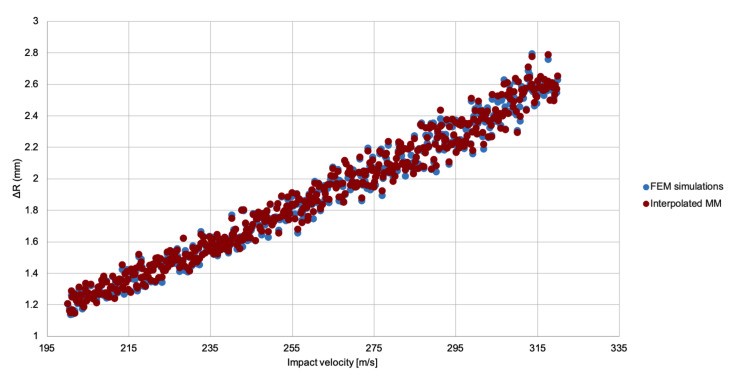
Comparison of meta-models interpolated results and FEM simulation results, considering ΔR for the Zerilli–Armstrong model.

**Figure 12:**
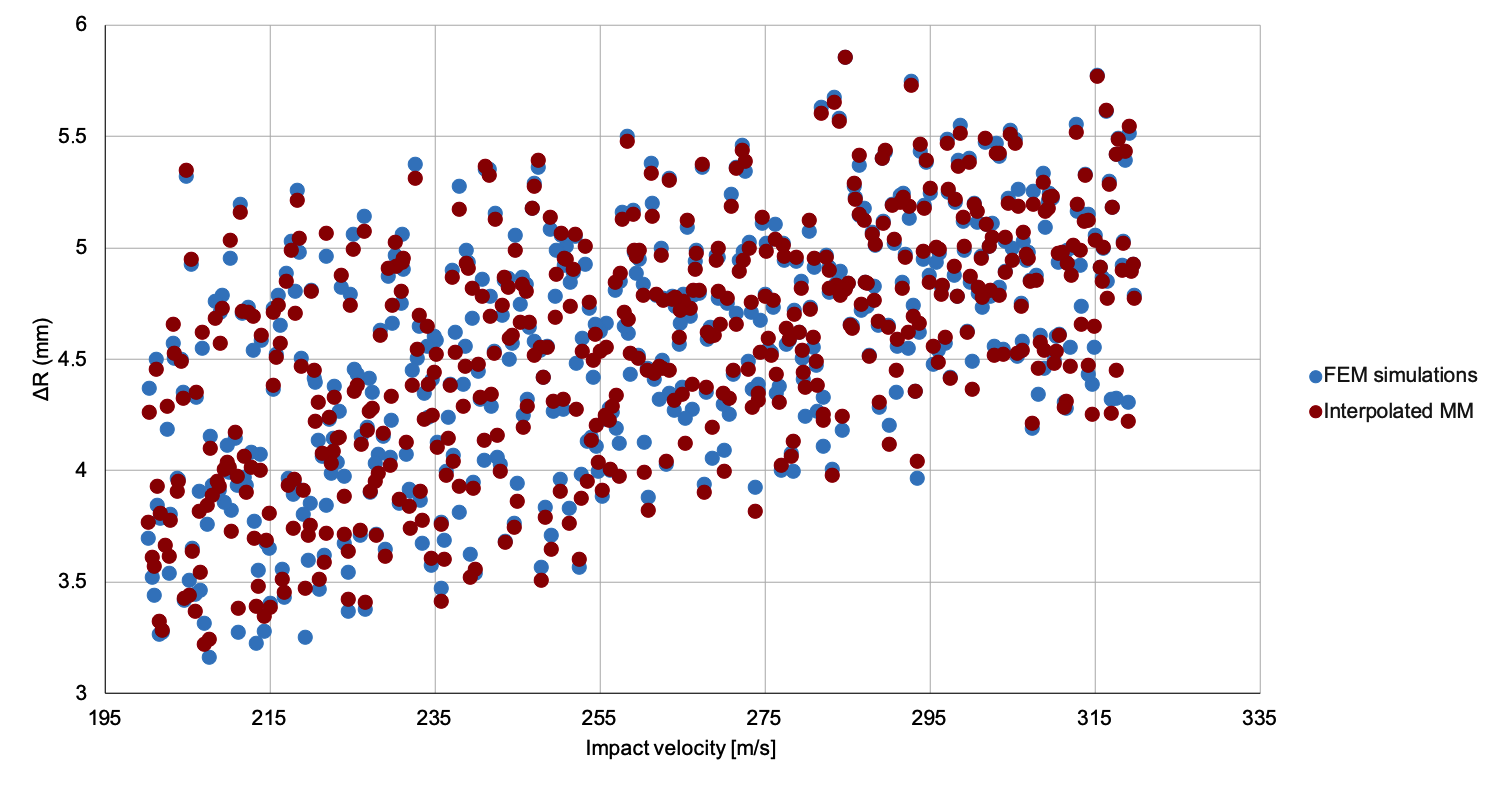
Comparison of meta-models interpolated results and FEM simulation results, considering ΔR for the Arrhenius-type model.

In Section 3, it was proposed to interpolate linearly meta-model predictions to extend the latter to arbitrary anvil velocities. Next, in Figure 10, Figure 11 and Figure 12, we show a comparison between the predictions of the QoI ΔR obtained from meta-model interpolation and full FE simulations.

Observing these plots and the results collected in Table 4, we conclude that the meta-model interpolation for the Johnson–Cook and Zerilli–Armstrong models provides accurate predictions of ΔR for arbitrary impact velocities. In contrast, the interpolations of the Arrhenius-type model are not as accurate, possibly due to the relatively higher non-linearity of its constitutive equation, affecting directly the flow stress computation. Without a direct means of verifying this assertion, we might speculate that these non-linearities trigger complex deformation patterns in the anvil once the material enters the plastic regime. However, given that the maximum relative error is below $7\times 10^{-2}$ in all three cases, we can accept the interpolated predictions for the three constitutive models. This choice will result in huge computational savings for the Bayesian calibration.

We have also validated the linear interpolation strategy for the quantity ΔL. The results are very similar to the ones obtained for ΔR and the interpolation plots are not presented. Table 5 collects the errors made by the meta-model for ΔL as compared with the FE solution, leading us to conclude, as for the previous QoI, that the interpolated predictions are accurate enough.

### 4.3. Bayesian Calibration

**Figure 13:**
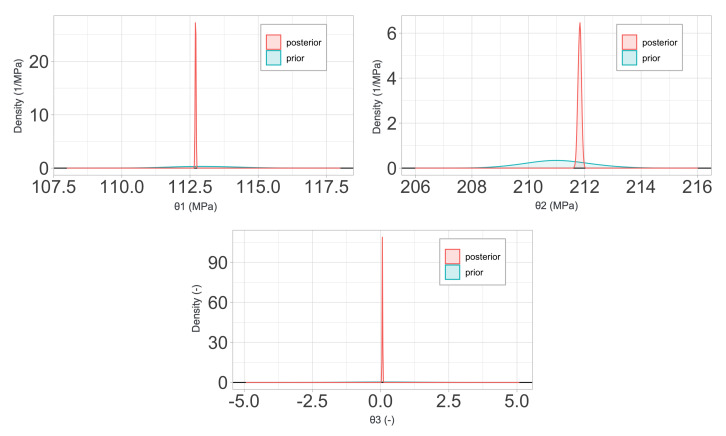
Prior vs. posterior probability distribution functions of parameters $A∼\theta_{1},B∼\theta_{2},C∼\theta_{3},$ in the JC model considering ΔR as the QoI.

**Figure 14:**
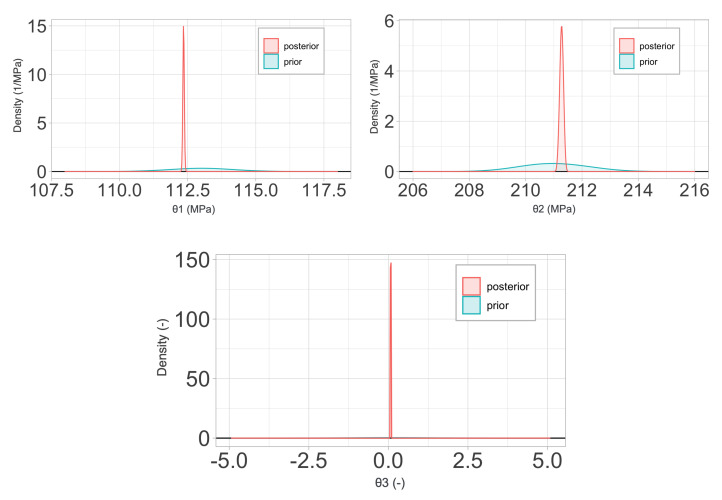
Prior vs. posterior probability distribution functions of parameters $A∼\theta_{1},B∼\theta_{2},C∼\theta_{3},$ in the JC model considering ΔL as the QoI.

**Figure 15:**
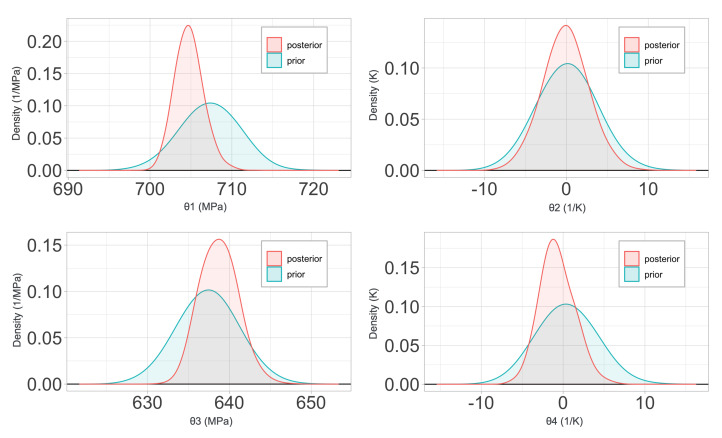
Prior vs. posterior parameter probability distributions for $C_{0}∼\theta_{1},C_{3}∼\theta_{2},C_{5}∼\theta_{3},n∼\theta_{4}$, of the ZA model considering ΔR as the QoI.

**Figure 16:**
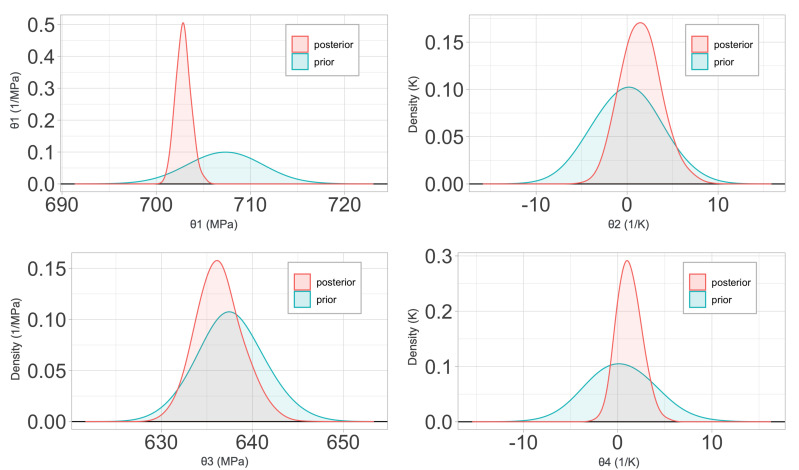
Prior vs. posterior parameter probability distributions for $C_{0}∼\theta_{1},C_{3}∼\theta_{2},C_{5}∼\theta_{3},n∼\theta_{4}$, of the ZA model considering ΔL as the QoI.

**Figure 17:**
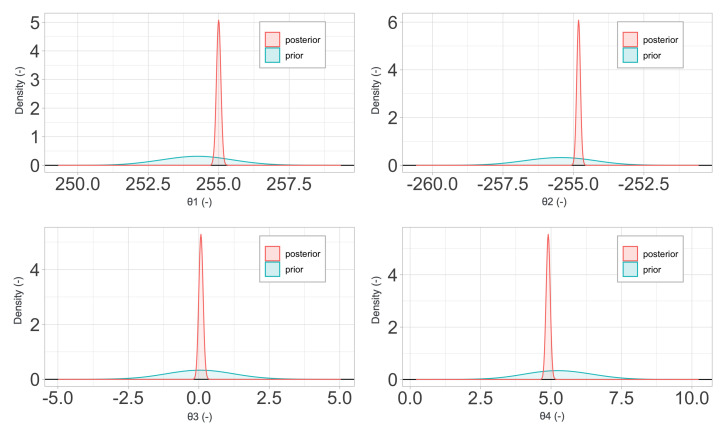
Prior vs. posterior parameter probability distributions for parameters $A_{2}∼\theta_{1},A_{3}∼\theta_{2},\alpha_{3}∼\theta_{3},n∼\theta_{4}$ of the Arrhenius-type model considering ΔR as the QoI.

**Figure 18:**
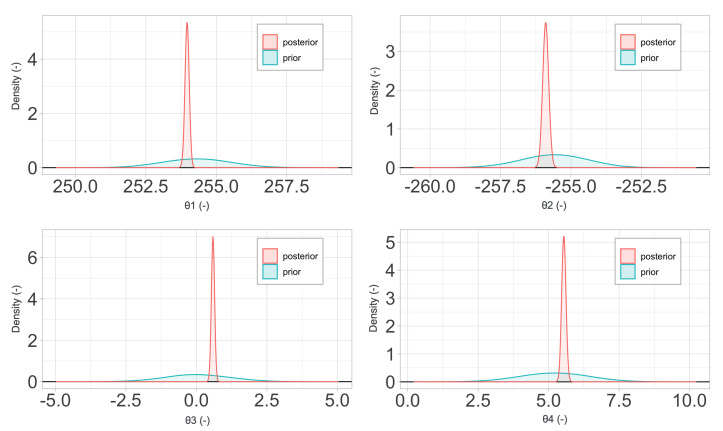
Prior vs. posterior parameter probability distributions for parameters $A_{2}∼\theta_{1},A_{3}∼\theta_{2},\alpha_{3}∼\theta_{3},n∼\theta_{4}$ of the Arrhenius-type model considering ΔL as the QoI.

Finally, we proceed to perform a Bayesian calibration of the material models employed in the sensitivity analysis, keeping fixed at their nominal value those parameters that have been found to be non-influential in the sensitivity analyses. The calibration results considering both QoIs, ΔR and ΔL, are shown in Figure 13, Figure 14, Figure 15, Figure 16, Figure 17 and Figure 18.

Specifically, Figure 13 shows the prior probability distribution functions provided for the three most relevant parameters, A,B, and *C* of the JC model, and their posterior probability functions. Similarly, Figure 15 illustrates the same probability functions, now for the most relevant parameters of the ZA model: namely, $C_{0},C_{3},C_{5}$, and *n*.

Based on the results of the calibration, we can make general comments on the calibrated models. In the case of the JC constitutive law, the calibration process has notably sharpened the probability density function of the three most significant parameters (see Figure 13 and Figure 14), eliminating a great part of the uncertainty linked to the variance in the prior probability distributions. Comparing the calibrated values of the parameters in the JC model obtained for the two QoIs analyzed (see Table 6), we note that both are similar. This suggests that the JC model is a good constitutive model for capturing the physics behind Taylor’s test. In turn, the posterior probability distributions for the ZA model barely reduce the variance of the priors (cf. Figure 15 and Figure 16). As a consequence, the calibration does not reduces significantly the uncertainty in the parameters. In addition, some of the calibrated parameters for the two QoIs under study have large disparities in their means. This is a consequence of the fact that, in our experiments, simulations carried out with the ZA model predict softer results, irrespective of the impact velocity and QoI observed. This fact, far from being a negative result, proves the potential of the method and illustrates that when the experimental and the simulation data are not in full agreement, the outcome of the calibration alerts of more uncertainties in the model and/or the data, or even the inability of the constitutive model to capture the physics of the problem.

Regarding the calibration of the Arrhenius model, it can be observed from Figure 17 and Figure 18 that the variance reduction in the posterior probability distribution of its parameters is not as strong as for the Johnson–Cook constitutive law, although it is still significant when compared to the Zerilli–Armstrong case. Something similar happens when analyzing the (mean) calibrated parameters when considering the two QoIs. Even if there is a good agreement among them, the calibrated parameter $\alpha_{3}$ obtained for the two QoI is fairly different. A potential explanation can be found, again, in the complexity of the constitutive equation and the effects of ignoring a large number of the model parameters. While this conscious choice saves much computational cost, it causes the loss of information in the model behavior, that, either way, could be countered to a large extent with additional simulated data at a low computational cost.

## 5. Conclusions

Calibrating complex material models using experimental tests and simulations is a critical task in computational engineering. When done in combination with statistical inference, this process can yield accurate values for the unknown material parameters plus additional information about its scatter and intervals of confidence. For example, Gaussian processes provide a natural and powerful framework to combine physical and numerical tests to obtain probability distributions of the material parameters. To fully exploit the potential of this kind of analysis, however, a large number of data points is required, and the latter can be most effectively obtained by employing a meta-model.

In this work, we described an effective framework for calibrating complex material models based on the combination of meta-models built on top of anisotropic Radial Basis Functions, Global Sensitivity Analysis, and Gaussian processes. The integration of these techniques results in a robust and efficient workflow.

We have employed the framework described for the calibration of three extremely common, although complex material models. These are the Johnson–Cook, the Zerilli–Armstrong, and Arrhenius-type models, and are typically employed for the characterization of the elasto-visco-plastic response of metals under high strain rates, and possibly high temperature as well. The outcome of our analysis is two-fold. First, we are able, for each material model, to rank the sensitivity of an impact simulation with respect to each of the parameters involved. Second, the framework produces a probability distribution for all the calibrated parameters as a function of the available or generated data, tapping into previously built and extremely fast statistical tools to obtain them. Such a characterization is more complete than simple point estimates, often employed when fitting material models.

Let us conclude by noting that the procedures described in this work have applicability beyond materials calibration to, in principle, problems where model evaluations and experimental setups are costly.

## Figures and Tables

**Table:** 

0.48	0.48	0.48
(a)	(b)	(c)

**Table:** 

0.48	0.48	0.58
(a)	(b)	(c)

**Table:** 

0.48	0.48	0.48	0.48
(a)	(b)	(c)	(d)

**Table:** 

0.48	0.48	0.48	0.48
(a) h	(b) h	(c) h	(d) h

**Table:** 

0.48	0.48	0.48	0.48
(a)	(b)	(c)	(d)

**Table:** 

0.48	0.48	0.48	0.48
(a) h	(b) h	(c) h	(d) h

**Table 1:** Prior probability distributions.

Term/Parameter	Probability Distribution Function
θ	N(μ,1) JC/ARR or N(μ,10) ZA (see Table 2)
$\lambda^{2}$	Γ(1,0.1)
$\sigma_{\delta }^{2}$	Γ(1,0.1)
$\psi_{\delta }$	U(0,1)

**Table 2:** Mean values for the parameter distributions according to literature [19,33].

Material Model	Mean Values
Johnson–Cook	A=113MPa,B=211MPa,C=0.073,n=0.218,m=0.818
Zerilli–Armstrong	$C_{0}=707.2\;\mathrm{MPa},\;C_{1}=575\;\mathrm{MPa},\;C_{3}=0.00698\;K^{-1},\;$
	$C_{4}=0.00032\;K^{-1},\;C_{5}=637.5\;\mathrm{MPa},\;n=0.41$
Arrhenius-type	$Q_{0}=412.31,\;Q_{1}=-510.82,\;Q_{2}=1873.4,\;Q_{3}=-1872.4$
	$A_{0}=36.402,\;A_{1}=-68.301,\;A_{2}=254.32,\;A_{3}=-255.57$
	$\alpha_{0}=0.009481,\;\alpha_{1}=-0.003841,\;\alpha_{2}=-0.012971,\;\alpha_{3}=0.025892,\;$
	n=5.2248

**Table 3:** Model parameters accounting for 90% or more of the Quantity of Interest (QoI) variance.

Material Model	Significant Parameters
Johnson–Cook	A,B,C
Zerilli–Armstrong	$C_{0},C_{3},C_{5},n$
Arrhenius-type	$A_{2},A_{3},\alpha_{3},n$

**Table 4:** Errors in the meta-model predictions of ΔR compared with full FE simulations.

Model	Mean Relative Error	Maximum Relative Error
Jonhson–Cook	$6.2\times 10^{-3}$	$1.2\times 10^{-2}$
Zerilli–Armstrong	$8.1\times 10^{-3}$	$3.2\times 10^{-2}$
Arrhenius-type	$1.1\times 10^{-2}$	$6.7\times 10^{-2}$

**Table 5:** Errors in the meta-model predictions of ΔL compared with full FE simulations.

Model	Mean Relative Error	Maximum Relative Error
Jonhson–Cook	$6.9\times 10^{-3}$	$2.3\times 10^{-2}$
Zerilli–Armstrong	$9.4\times 10^{-3}$	$3.6\times 10^{-2}$
Arrhenius-type	$7.8\times 10^{-3}$	$3.0\times 10^{-2}$

**Table 6:** A posteriori mean values obtained for each parameter, considering both QoIs, ΔR and ΔL, for the calibration of the models.

Material Model	Mean Values ΔR	Mean Values ΔL
Johnson–Cook	A=112.7MPa,B=211.8MPa,C=0.065	A=112.4MPa,B=211.3MPa,C=0.073
Zerilli–Armstrong	$C_{0}=704.6\;\mathrm{MPa},\;C_{3}=-0.00392\;K^{-1},\;$	$C_{0}=702.8\;\mathrm{MPa},\;C_{3}=1.65\;K^{-1},\;$
	$C_{5}=638.3\;\mathrm{MPa},\;n=-1.65$	$C_{5}=636.1\;\mathrm{MPa},\;n=1.02$
Arrhenius-type	$A_{2}=255.0,\;A_{3}=-254.8$	$A_{2}=254.0,\;A_{3}=-255.9$
	$\alpha_{3}=0.077,\;n=4.9$	$\alpha_{3}=0.59,\;n=5.54$
